# Supplementary figures and images for: Drug Resistance in Glioma Cells Induced by a Mesenchymal–Amoeboid Migratory Switch
Source: Biomedicines. 2021 Dec 22;10(1):9. doi: 10.3390/biomedicines10010009 (PMC8773151; doi:10.3390/biomedicines10010009)

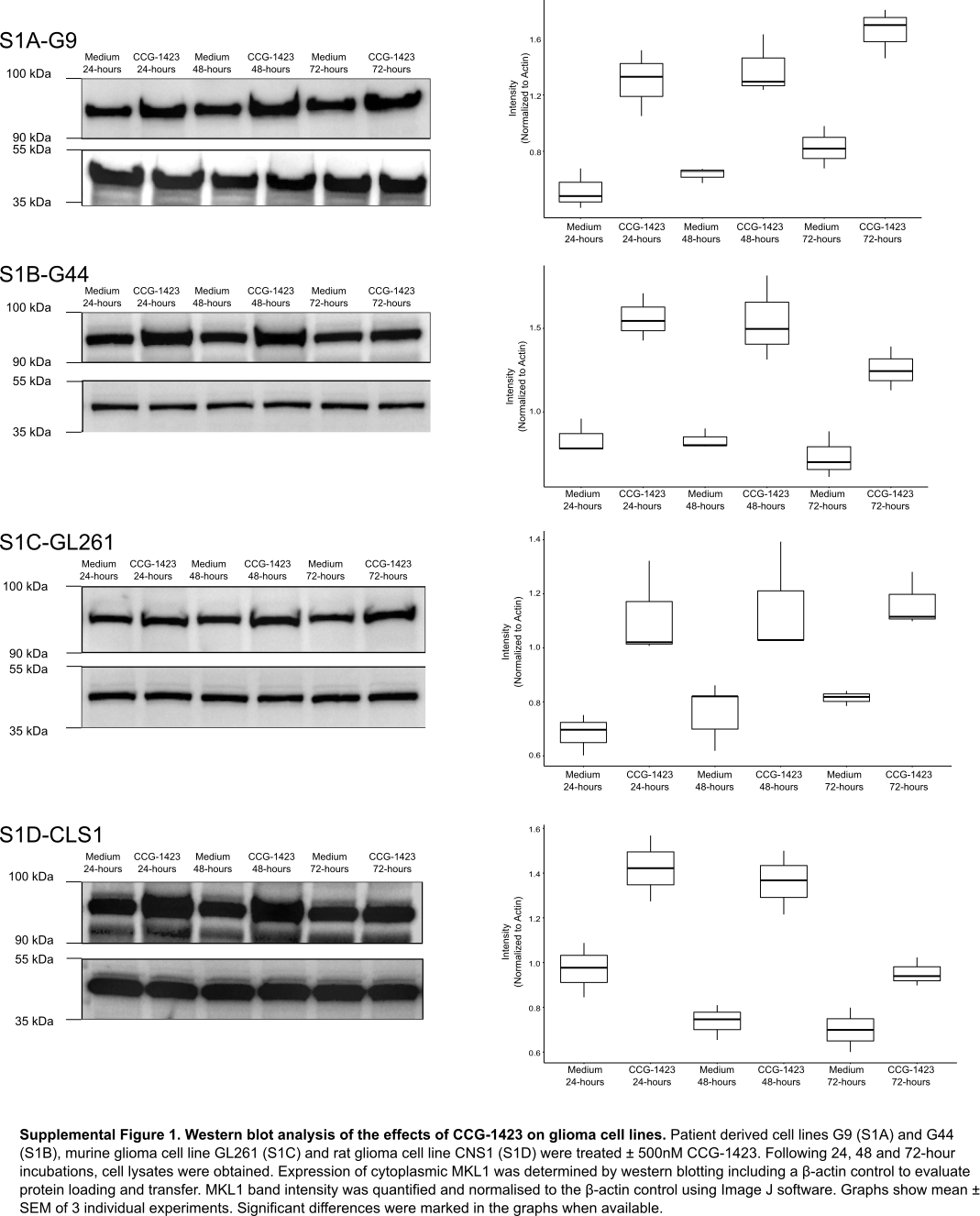

Supplement: Supplementary file 1 [file biomedicines-10-00009-s001.zip › biomedicines-1397725-supplementary/New folder/Supplement1.png]

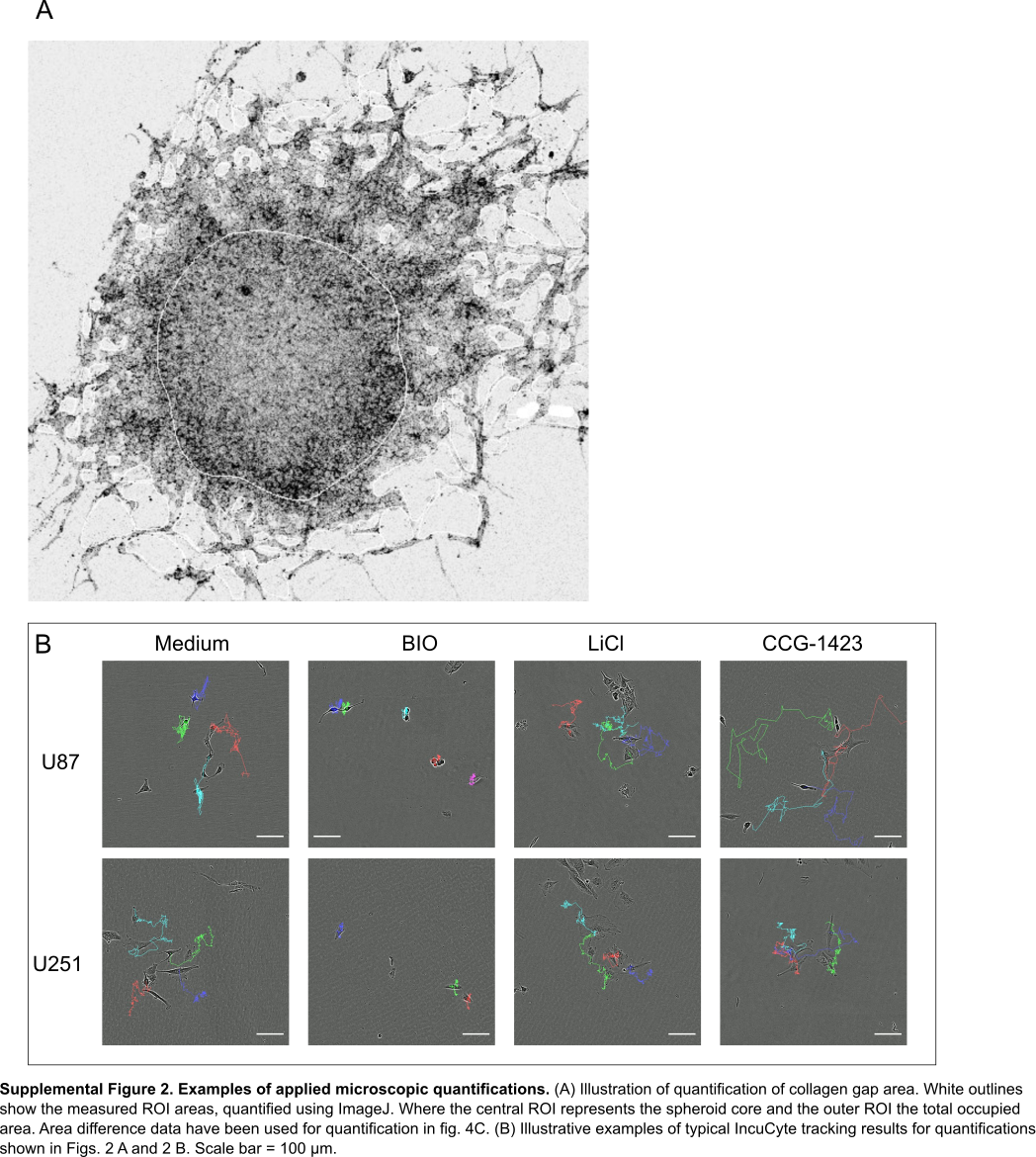

Supplement: Supplementary file 1 [file biomedicines-10-00009-s001.zip › biomedicines-1397725-supplementary/New folder/Supplement2_plus_tracks.png]
